# Notes and News

**Published:** 1896-05-23

**Authors:** 


### Notes and News.

(Collected and Communicated.)

### HOSPITAL MEETINGS.

The Annual General Court of the Governors of the Hospital for Sick Children, Great Ormond Street, W.C., will be held in the Board Room of the Hospital on Friday, May 29th, at 4.30 p.m.

The committee of the Paddington Green Children's Hospital have received a donation of £1,000 from the executrix of the late Mrs. Juliana Josephine McAlpine, for a cot, to be named "Margaret Charlotte McAlpine."

The trustees of the Earl of Moray have allocated the sum of £40,000 left under his will for charitable and beneficent institutions in Moray, Nairn, Inverness, Fife, and Perth. Amongst the allocations are: Northern Infirmary, Inverness, £4,000; Highland Orphanage, Inverness, £2,000; Blind Asylum, Inverness, £200; Perth Infirmary, £2,500; Convalescent Home, Nairn, £300; Nairn Hospital, £200; Leanchoil Hospital, Forres, £700; Gray's Hospital, Elgin, £2,500; Indigent Gentlewomen's Fund, £500; Dispensary Fund, £500.

It is always gratifying when we find our efforts on behalf of the hospitals are appreciated. We have read, therefore, with much pleasure the following paragraph which appears in the annual report of the Westminster Hospital, one of the oldest and most economically-administered hospitals in London: "The total of the Sunday Fund collection was largely in excess of that of any previous year, a result in a great measure attributable to the energetic and self-denying efforts of Mr. Henry C. Burdett. The Committee are glad to embrace this opportunity of recording their grateful appreciation of Mr. Burdett's long devotion to the cause of hospitals, and his many years of successful labour on their behalf."

The *Yale Medical Journal*, commenting on the Kitson v. Playfair case, mentions that in New York State a physician is not compelled to divulge any professional secrets even in a court of law.

Close to London, only about six miles from the Tower Bridge, there lies buried out of sight one of the most wonderful results of engineering skill which this century has produced. The Blackwall Tunnel is now nearly finished, and by the courtesy of Sir Weetman Pearson, Bart., M.P. for Colchester, the head of the firm by which the work has been carried out for the County Council, it will be thrown open for the inspection by visitors at Whitsuntide, for the benefit of the Seamen's Hospital Society. There will be much more to see then than there will later when it is formally opened for the use of the public, for the ponderous shields by which the tunnel has been driven through the bed of the river will be on view, and visitors will be able to form some idea of the method by which great works of this sort have to be carried on. All the vast machinery used in the construction of the tunnel will be on view and in working order, and it should form a most interesting exhibition.

Saturday, the 16th inst., was the first "Lifeboat Saturday" in London. Besides an organised system of street collections, large processions paraded various parts of the metropolis. As we have pointed out on many previous occasions, street collections are wrong in principle, and tend to defeat the object for which they are supposed to be made, viz., the bringing in of funds for charitable purposes. Leaving out the abuses to which they are open, the business man objects to being pestered at every corner on the way to his office, whether by ladies, men in labourers' clothes, or boys in sailors' costumes. The consequence is his annoyance prevents his giving liberally when afterwards approached in a legitimate way. A procession, too, half a mile long, as one was on Saturday, upsetting the traffic in a crowded district, cannot fail to discredit any cause, however good in itself. We have little doubt that this degenerate departure on the part of its managers will cost the Royal National Lifeboat Institution a loss of income to the extent of some thousands a year. We hope therefore that this may prove the last as it was the first Lifeboat Saturday in London.

How to get governors and subscribers to attend the annual meeting is a question which greatly exercises the minds of London hospital secretaries. For instance, at a meeting we recently attended, although 4,000 notices were sent out to the governors, and in addition the meeting was advertised in the daily papers, only five persons put in an appearance, the majority of whom were members of the board of management. London may sometimes learn something from the provinces, and it may be useful to state what has been done at a provincial institution which found itself a year or two back in the same predicament as the metropolitan hospitals. Determined to rouse the flagging interest in their annual meeting, the Wolverhampton and District Hospital for Women hit on the plan of making that meeting a sort of "at home," at which, in addition to the ordinary business, an excellent concert was given by the friends of the hospital. This scheme has worked most successfully, the attendance being always very large, and the interest taken in the hospital is proportionately increased.

